# Neurofilament-Light Chain and Glial Fibrillary Acidic Protein as Blood-Based Delirium Risk Markers: A Multicohort Study

**DOI:** 10.14336/AD.2025.0107

**Published:** 2026-04-18

**Authors:** Maria Cristina Ferrara, Lucía Lozano-Vicario, Beatrice Arosio, Cristina D’Orlando, Lara De Luca, Alice Margherita Ornago, Elena Pinardi, Paolo Mazzola, Chukwuma Okoye, Riccardo Gamberale, Francesca Remelli, Massimiliano Castellazzi, Giovanni Zatti, Giuseppe Foti, Ángel Javier Muñoz-Vázquez, Nicolás Martínez-Velilla, Stefano Volpato, Giuseppe Bellelli, on behalf of the ORTODEL group (Appendix 1)

**Affiliations:** ^1^School of Medicine and Surgery, University of Milano-Bicocca, 20900 Monza, Italy.; ^2^Department of Geriatric Medicine, Hospital Universitario de Navarra (HUN), 31008 Pamplona, Spain.; ^3^Department of Clinical Sciences and Community Health, University of Milan, 20122 Milan, Italy.; ^4^Department of Pathophysiology and Transplantation, University of Milan, 20122 Milan, Italy.; ^5^Acute Geriatric and Orthogeriatric Unit, IRCCS Foundation San Gerardo dei Tintori, 20900 Monza, Italy.; ^6^Department of Medical Sciences, University of Ferrara, 44121 Ferrara, Italy.; ^7^Department of Neurosciences and Rehabilitation, University of Ferrara, 44121 Ferrara, Italy.; ^8^Orthopedic Unit, IRCCS Foundation San Gerardo dei Tintori, 20900 Monza, Italy.; ^9^Anesthesiology Unit, IRCCS Foundation San Gerardo dei Tintori, 20900 Monza, Italy.; ^10^Department of Orthopaedic Surgery and Traumatology, Clínica Universidad de Navarra, 31008 Pamplona, Spain.; ^11^Navarrabiomed, Hospital Universitario de Navarra (HUN), Universidad Pública de Navarra (UPNA), Instituto de Investigación Sanitaria de Navarra (IdiSNA), 31008 Pamplona, Spain.; ^12^CIBER of frailty and healthy aging (CIBERFES), Instituto de Salud Carlos III, 28029 Madrid, Spain.

**Keywords:** delirium, blood-based biomarker, neurofilament-light chain, glial fibrillary acidic protein, hip fracture

## Abstract

Postoperative delirium (POD) is often under-recognized in clinical practice. Blood-based markers could facilitate delirium detection and risk stratification. Neurofilament-Light chain (NfL) and Glial Fibrillary Acidic Protein (GFAP) are promising blood-based markers for neurodegenerative diseases and potential candidates for delirium. This study explored their role as blood-based risk markers for POD in older patients undergoing hip fracture surgery. In this prospective multicohort study, preoperative blood and intraoperative cerebrospinal fluid (CSF) samples were collected from patients aged ≥65 years with hip fractures. POD was assessed daily using the 4AT scale. NfL and GFAP concentrations in both blood and CSF were compared between POD and Non-POD groups, further stratifying by dementia status. Logistic regression models adjusted for covariates were used to assess associations. A total of 143 patients (median age, 85 years; 76.9% female) were included, with POD occurring in 38 patients (26.6%). Blood NfL and GFAP concentrations were significantly higher in the POD group than in Non-POD (64.55 vs. 44.6 pg/mL and 22 vs. 14.8 pg/mL, P<0.001). CSF NfL levels were also elevated in POD (2154 vs. 1565 pg/mL, P=0.007), but no significant difference was observed for CSF GFAP levels. Higher preoperative blood NfL and GFAP levels independently predicted POD after adjusting for age, sex, dementia, frailty, and Interleukin-6 (Odds Ratio, OR: 3.21, 95% Confidence Interval, CI: 1.26-8.21, and OR: 3.66, 95% CI: 1.38-9.68, respectively). Although further research is needed, our findings support the role of NfL and GFAP as blood-based risk markers for POD in older patients undergoing hip fracture surgery.

## INTRODUCTION

Delirium is a neuropsychiatric disorder characterized by alterations in attention, cognitive dysfunction, and a fluctuating course that arises as a direct consequence of injury or acute medical illness [1].

It is a common complication in older patients undergoing surgery for hip fracture (HF) [2, 3], with a prevalence of postoperative delirium (POD) ranging from 13% to 56% [4, 5]. Delirium poses a remarkable health concern due to its association with several adverse outcomes, including patient and caregiver distress, increased healthcare costs, long-term cognitive and functional decline, and mortality [2, 3, 6, 7]. Despite its impact, the diagnosis of delirium remains challenging, with up to 70% of cases remaining undetected [8], leading to diminished care quality and outcomes [9]. Given this context, blood-based markers (BBMs) could play a crucial role in identifying at-risk patients and aiding in differential diagnoses in clinical practice [10]. Although current evidence does not support the use of any specific biomarker for delirium, certain markers have recently emerged with promising results [11–15].

Neurofilament-Light chain (NfL) is a scaffolding protein of the neuronal cytoskeleton, primarily located in myelinated axons, which provides increased conduction speed and structural support [16]. Glial fibrillary acidic protein (GFAP) is the key intermediate filament in mature astrocytes -equivalent to NfL in neurons-, responsible for organizing the cytoskeletal structure of glial cells and supporting neighbouring neurons [17]. Elevated levels of NfL and GFAP have been observed across a wide range of neurodegenerative disorders, including Alzheimer's disease and neuroperturbative conditions; however, the results remain inconsistent and non-univocal, warranting further investigation [18–23]. Given the bidirectional pathophysiological connections between dementia and delirium [24], novel insights into the neurodegenerative processes linked to delirium could stem from exploration of the roles of NfL and GFAP in the orthogeriatric setting.

This study aimed to investigate the potential role of NfL and GFAP as BBMs in the risk of POD in older patients undergoing HF surgery. Secondary objectives included assessing how dementia status influences the relationship between BBMs and POD, as well as investigating the correlations between NfL and GFAP concentrations in both the blood and CSF.

## MATERIALS AND METHODS

### Study participants

This study combined two prospective cohorts of inpatients, one Italian and one Spanish, using a similar study design. The Italian cohort (ORTODEL) enrolled patients consecutively admitted to Orthogeriatric Units in Monza and Ferrara from July 2021 to March 2024 [25]. The Spanish cohort (BIODEL) enrolled patients consecutively admitted to the Orthogeriatric Unit of the Hospital Universitario de Navarra (Pamplona) between August 2021 and December 2021 [26]. The variables used in this study were retrospectively harmonized according to current recommendations [27].

The eligibility criteria for the ORTODEL and BIODEL cohorts have been previously described elsewhere [25, 26]. Briefly, the eligibility criteria included patients aged ≥ 65 years who underwent surgical repair for acute HF. Patients were excluded from the analysis if they 1) had preoperative delirium, 2) had terminal disease with an estimated life expectancy of less than 3 months, 3) were unable to communicate, or 4) were unwilling or unable to provide informed consent.

### Standard protocol approvals, registrations, and patient consent

Written informed consent for study participation was obtained from patients at enrolment in both cohorts, according to procedures approved by the institutional review boards (Ethics Committee of Monza Brianza on May 6, 2021 [decree 691], study reference number 3505, and Navarra Clinical Research Ethics Committee on June 25, 2021 [PI_2021/68]).

### Clinical assessment and covariates

A comprehensive geriatric assessment was performed at the time of enrolment for both patient cohorts. Presurgical baseline variables were collected by interview or chart review, including age, sex, Charlson Comorbidity Index (CCI), diagnosis of dementia, and frailty assessed using the Clinical Frailty Scale. Diagnosis of dementia was established according to DSM-5 criteria [1] by an expert geriatrician at each center (LLZ in Pamplona, PM in Monza, FR in Ferrara). The diagnosis was based on a combination of anamnestic history, review of medical records, caregiver interviews using sensitive screening tests (i.e., AD8 [28] and IQ-CODE [29]), and medication use upon admission. Additionally, surgical variables such as the time to surgery and type of anaesthesia (general or spinal) were recorded.

### Outcome assessment

Delirium was assessed daily, preoperatively and for up to three days postoperatively, by geriatricians using the Spanish or Italian versions of the 4AT scale [30, 31]. The 4AT is a simple and quick delirium detection tool designed for clinical use that requires no special training. It has high sensitivity and specificity (both 88%) and its use as a diagnostic tool is supported by several studies [32–34]. The assessment was based on patient examination, review of medical and nursing records, and information provided by caregivers and next of kin. The final diagnosis of POD was confirmed by a senior geriatrician with expertise in delirium according to DSM-5 criteria (acute disturbance in attention and awareness, with fluctuations during the course of a day and representing a change from the patient’s baseline condition) [1].

### Specimen collection

Blood samples (4-5 mL) were collected preoperatively by venipuncture and processed within 1 h. Polypropylene tubes with EDTA were used for collection (vacuette). Blood samples were centrifuged at 2500 rpm for 10 min at 4°C to separate the plasma from cellular components. Aliquots of 0.5-1.0 mL were stored at −80°C.

Whenever possible, cerebrospinal fluid (CSF) sampling was performed in patients undergoing spinal anaesthesia by spinal cannulation under standard monitoring in the operating room immediately before the administration of anaesthetic agents for the planned surgical procedure. After confirming intrathecal placement and adequate CSF flow, 1.5 mL of CSF was collected by aspiration or directly dropwise into polypropylene tubes and stored at − 80 °C. Rigorous quality control standards were used to ensure the integrity of the ORTODEL and BIODEL biospecimens.

### Biomarker assay

Human Simple Plex assays (ProteinSimple, CA, USA) on an Ella device (Bio-Techne, MN, USA) were used to quantify blood and CSF concentrations of NfL, GFAP, and IL-6. Instrument calibration was performed using the cartridge factory standard curve, and blood/CSF samples were measured with dilution in Sample Diluent according to the manufacturer’s instructions (Bio-Techne, MN, USA). A single well was used for each sample, because triplicate assays were performed automatically using the Simple Plex assay microfluidic platform. All biological samples were analysed simultaneously in the same laboratory by technicians blinded to the clinical data.

**Table 1:** Characteristics of the study population, with or without postoperative delirium.

Variables^a^	Total (n=143)	Non-POD^b^ (*n*=105)	POD^b^ (*n*=38)	p-value
**Country**				0.052
**Italy**	83 (58)	66 (62.9)	17 (44.7)	
**Spain**	60 (42)	39 (37.1)	21 (55.3)	
**Age**	85 (79-89)	83 (77-88)	88.5 (85-91)	< 0.001
**Sex (female)**	110 (76.9)	77 (73.3)	33 (86.8)	0.090
**Dementia**	24 (16.8)	10 (9.5)	14 (36.8)	< 0.001
**Charlson Comorbidity Index score**	5 (4-6)	5 (4-7)	6 (4.7-6)	0.164
**Clinical Frailty Scale**	4 (3-6)	4 (3-5)	5 (3-6)	0.033
**Time to surgery (days)**	2 (1-2)	2 (1-2)	1 (1-2)	0.119
**General anaesthesia**	11 (7.7)	10 (11)	1 (2.6)	0.288
**Preoperative blood IL-6^c^ (pg/mL)**	36.8 (23-53.5)	38.5 (23.7-64.8)	34.6 (22.8-52.6)	0.534
**Preoperative blood NfL^d^ (pg/mL)**	48.9 (32.3-78.5)	44.6 (30.8-68.6)	64.5 (49.8-110.2)	< 0.001
**Preoperative blood GFAP^e^ (pg/mL)** ***(n=135)***	16.6 (10.9-23.9)	14.8 (9.2-21.2)	22 (17.3-28.7)	< 0.001
**CSF^f^ NfL^d^ (pg/mL) *(n=106)***	1725.5 (1270.7- 2169)	1565 (1169-2063)	2154 (1356-2773)	0.007
**CSF^f^ GFAP^e^ (pg/mL) *(n=104)***	581 (410 - 797)	576 (392-812)	585 (460-785)	0.543

a Nominal variables are expressed as n (%), while continuous variables are expressed as median (interquartile range);

b POD= postoperative delirium;

c IL-6= Interleukin-6;

d NfL = Neurofilament-Light chain;

e GFAP= Glial Fibrillary Acidic Protein;

f CSF = Cerebrospinal fluid.

**Figure 1.**
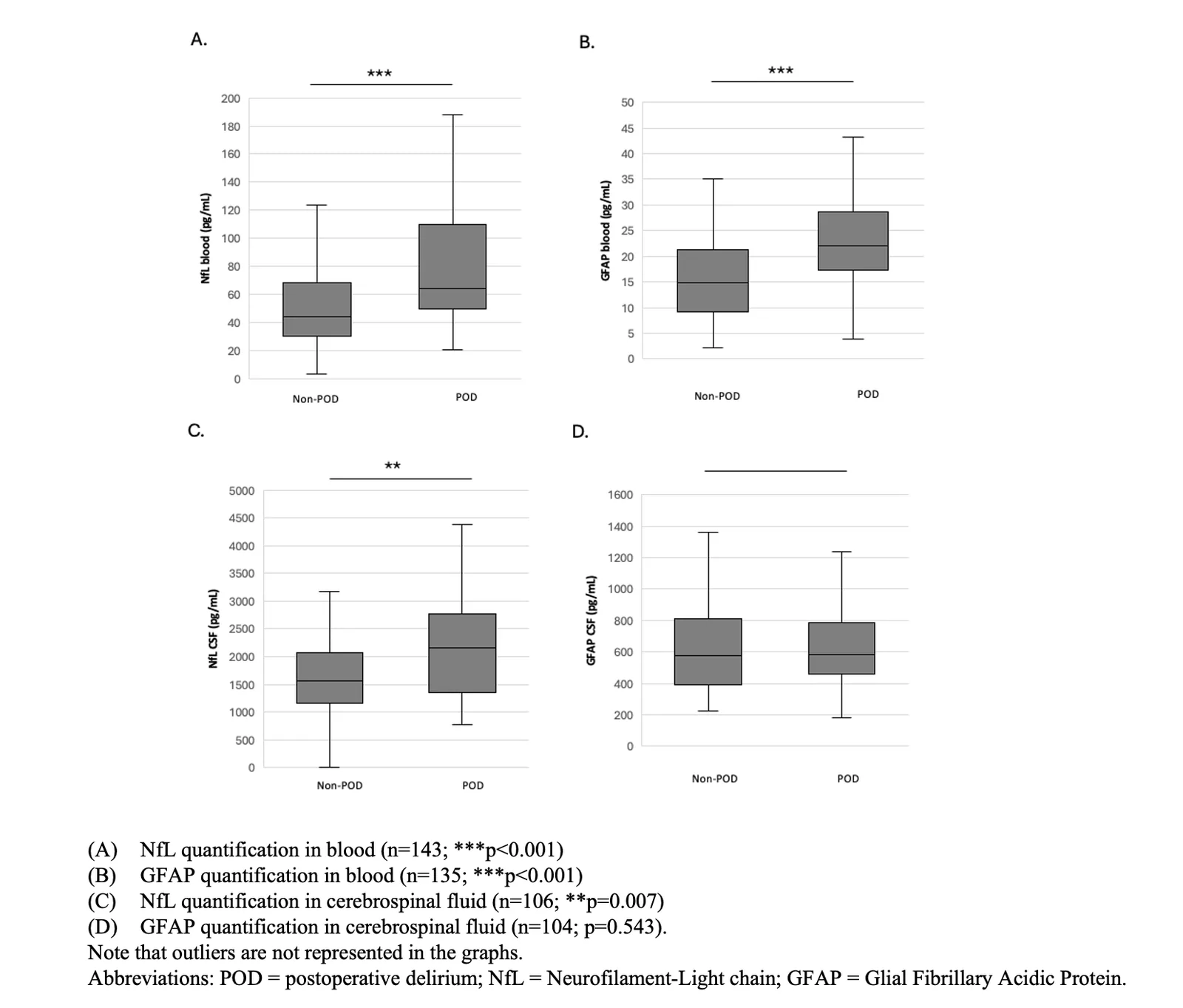
**NfL and GFAP concentrations in blood and CSF according to postoperative delirium (p-values refer to unadjusted comparison). (A)** NfL quantification in blood (n=143; ***p<0.001). **(B)** GFAP quantification in blood (n=135; ***p<0.001). **(C)** NfL quantification in cerebrospinal fluid (n=106; **p=0.007). **(D)** GFAP quantification in cerebrospinal fluid (n=104; p=0.543). Note that outliers are not represented in the graphs. Abbreviations: POD = postoperative delirium; NfL = Neurofilament-Light chain; GFAP = Glial Fibrillary Acidic Protein.

### Statistical analysis

The description of the sample according to the incidence of POD was conducted using medians and interquartile ranges for quantitative variables and frequencies and percentages for qualitative variables. Normality was checked for each variable using the Kolmogorov-Smirnov test and normal probability plots. The Mann–Whitney U-test was used to compare continuous variables between groups. The chi-squared test or Fisher’s exact test was used for categorical variables. The Kruskal-Wallis test was used for comparisons involving more than two patient groups (stratified by the presence or absence of delirium and dementia), with the Bonferroni correction applied for multiple comparisons.

Linear regression analyses were conducted to isolate the effect of age on natural log-transformed biomarker concentrations, followed by t-tests to compare the residuals between POD and Non-POD groups. Then, a baseline logistic regression model adjusted for age and sex was performed using natural log-transformed BBMs to evaluate their continuous effect on POD. Subsequently, NfL and GFAP concentrations were dichotomized at their median values to better address data skewness and minimize the impact of extreme values. NfL and GFAP concentrations were therefore treated as categorical dependent variables in the fully adjusted logistic regression analyses. These multivariable analyses were conducted to evaluate the independent association of NfL and GFAP with POD, adjusting for clinically based confounders (age, sex, dementia, frailty) and preoperative blood Interleukin-6 (IL-6) levels to account for the potential role of systemic inflammation in neuronal injury [35, 36]. This approach allows for more robust comparisons between high- and low-concentration groups and simplifies the interpretation of results by making the findings more applicable to clinical decision-making. Spearman’s correlation was used to test the correlation between blood and CSF concentrations of both NfL and GFAP in the overall population and within the Non-POD or POD groups. Statistical analyses were performed using SPSS 26.0 (IBM Corp.) and Stata 18.0.

## RESULTS

### Characteristics of the study population

Patient characteristics are presented in Table 1. Overall, 143 patients were included in this study: 83 in the ORTODEL cohort and 60 in the BIODEL cohort (Supplementary Table 1 contains key demographic and clinical differences between the two cohorts). POD occurred in 38 (26.5 %) patients, who were older, frailer, and more frequently affected by dementia. The median CCI of the whole study sample was 5 (IQR 4-6), with no significant differences according to POD incidence. The patients underwent hip surgery after a median of 2 days (IQR 1-2) from hospital admission, and 132 (92.3%) under spinal anaesthesia. Blood samples were available for all patients, while CSF samples were available for 106 of the 143 patients. Supplementary Figure 1 shows a flowchart of the samples available for NfL and GFAP analyses in the blood and CSF.

**Figure 2.**
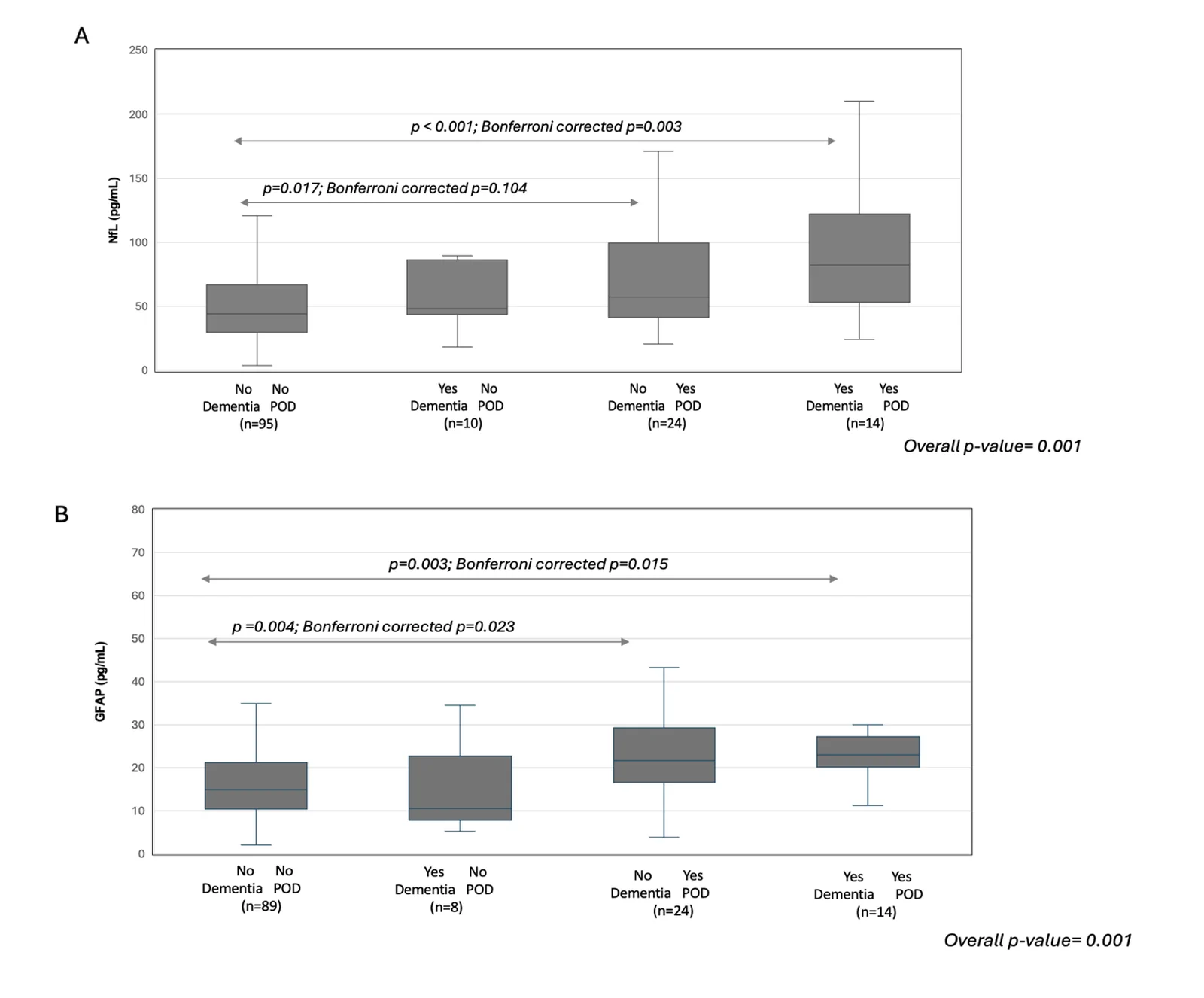
**NfL and GFAP concentrations in blood according to postoperative delirium and dementia status** (**p-values refer to unadjusted comparison)**. (**A**) NfL (**B**) GFAP. Abbreviations: POD = postoperative delirium; NfL = Neurofilament-Light chain; GFAP = Glial Fibrillary Acidic Protein.

**Table 2:** Association of NfL (Model A) and GFAP (Model B) with postoperative delirium.

Model A	OR^a^	95% C.I.^b^		p-value
Preoperative blood NfL^c^ (> median)	3.21	1.26	8.21	0.015
Age	1.09	1.02	1.17	0.017
Sex (female)	2.41	0.76	7.71	0.137
Dementia	4.88	1.58	15.04	0.006
Clinical Frailty Scale	0.93	0.69	1.26	0.641
Preoperative blood IL-6^d^ (> median)	0.91	0.38	2.22	0.844
Model B	OR^a^	95% C.I.^b^		p-value
Preoperative blood GFAP^e^ (> median)	3.66	1.38	9.68	0.009
Age	1.06	0.98	1.14	0.120
Sex (female)	2.07	0.64	6.67	0.224
Dementia	6.03	1.91	19.01	0.002
Clinical Frailty Scale	0.98	0.73	1.33	0.921
Preoperative blood IL-6^d^ (> median)	1.01	0.42	2.44	0.985

a OR= Odds Ratio;

b 95% C.I.=95% Confidence Interval;

c NfL= Neurofilament-Light chain;

d IL-6= Interleukin-6;

e GFAP= Glial Fibrillary Acidic Protein

### NfL and GFAP concentrations in blood and CSF according to POD

Figure 1 shows the distribution of NfL and GFAP concentrations in blood and CSF according to POD status. The median concentrations of NfL and GFAP in preoperative blood were significantly higher in the POD group than in the Non-POD group (64.55 pg/mL vs. 44.6 pg/mL and 22 pg/mL vs. 14.8 pg/mL, respectively; *P*<0.001). In addition, the median concentration of NfL in the CSF was significantly higher in the POD than in the Non-POD group (2154 pg/mL vs. 1565 pg/mL; *P*=0.007), while the GFAP concentration in the CSF did not significantly differ between the two groups.

Figure 2 shows the distribution of preoperative blood NfL concentrations according to POD and dementia status. A significant difference in NfL and GFAP blood concentrations was observed across the four groups (overall *P*=0.001). Notably, pairwise comparisons demonstrated that patients with both dementia and POD had significantly higher values of NfL and GFAP than those without dementia or POD. Furthermore, patients with POD but not dementia had higher values of both BBMs than those with neither dementia nor POD, although statistical significance remained only for GFAP after correction for multiple comparisons.

Supplementary Fig. 2 illustrates the distribution of NfL and GFAP concentrations in blood and CSF across different age categories in the POD and Non-POD groups. While linear regression revealed a significant association between age and BBMs, a comparison of residuals between the Non-POD and POD groups showed significant differences in preoperative blood levels of both NfL (*P* = 0.012) and GFAP (*P* = 0.024). These findings confirm that BBMs are significantly associated with POD, independent of age. Conversely, age was not significantly associated with CSF NfL levels in the linear regression analysis. Lastly, the linear regression residuals for CSF GFAP concentrations did not differ significantly between the POD and Non-POD groups, consistent with the non-significant findings presented in panel D of Figure 1.

Logistic regression models performed using natural log-transformed NfL and GFAP concentrations in preoperative blood showed that higher BBMs were significantly associated with an increased likelihood of POD, independent of age and sex (Odds Ratio, OR 2.19; 95% Confidence Interval, CI 1.11-4.32; OR 1.73; 95% CI 1.02-2.95, respectively). Table 2 shows the results of the fully adjusted multivariable logistic regression models for the likelihood of developing POD based on dichotomized blood-based NfL and GFAP concentrations (as separate models), adjusting for age, sex, dementia, Clinical Frailty Scale, and preoperative blood IL-6. Higher concentrations of NfL and GFAP in preoperative blood were significantly associated with an increased likelihood of POD (OR 3.21; 95% Confidence Interval, CI 1.26-8.21; OR 3.66; 95% CI 1.38-9.68, respectively). No statistically significant interactions between BBMs and dementia were detected (*P*>0.1, data not shown).

### Correlations between NfL and GFAP concentrations

Overall, significant positive correlations were observed between blood and CSF concentrations of both NfL (0.389, *P* <0.001) and GFAP (0.326, *P* <0.001), as well as between NfL and GFAP concentrations in both blood and CSF (0.502, *P* <0.001; 0.325, *P* <0.001) (Supplementary Fig. 3). After stratification according to POD status, NfL concentrations in the blood and CSF were significantly correlated in both groups (Non-POD 0.252, *P*=0.025; POD 0.560, *P*=0.002), as were NfL and GFAP concentrations in the blood (Non-POD 0.448, *P*<0.001; POD 0.454, *P*=0.004). In contrast, a significant correlation between blood and CSF concentrations of GFAP was observed only in the Non-POD group (0.364, *P*=0.001). Additionally, CSF concentrations of NfL and GFAP were significantly correlated only in the Non-POD group (0.364, *P*=0.001) (Supplementary Fig. 4).

**Figure 3.**
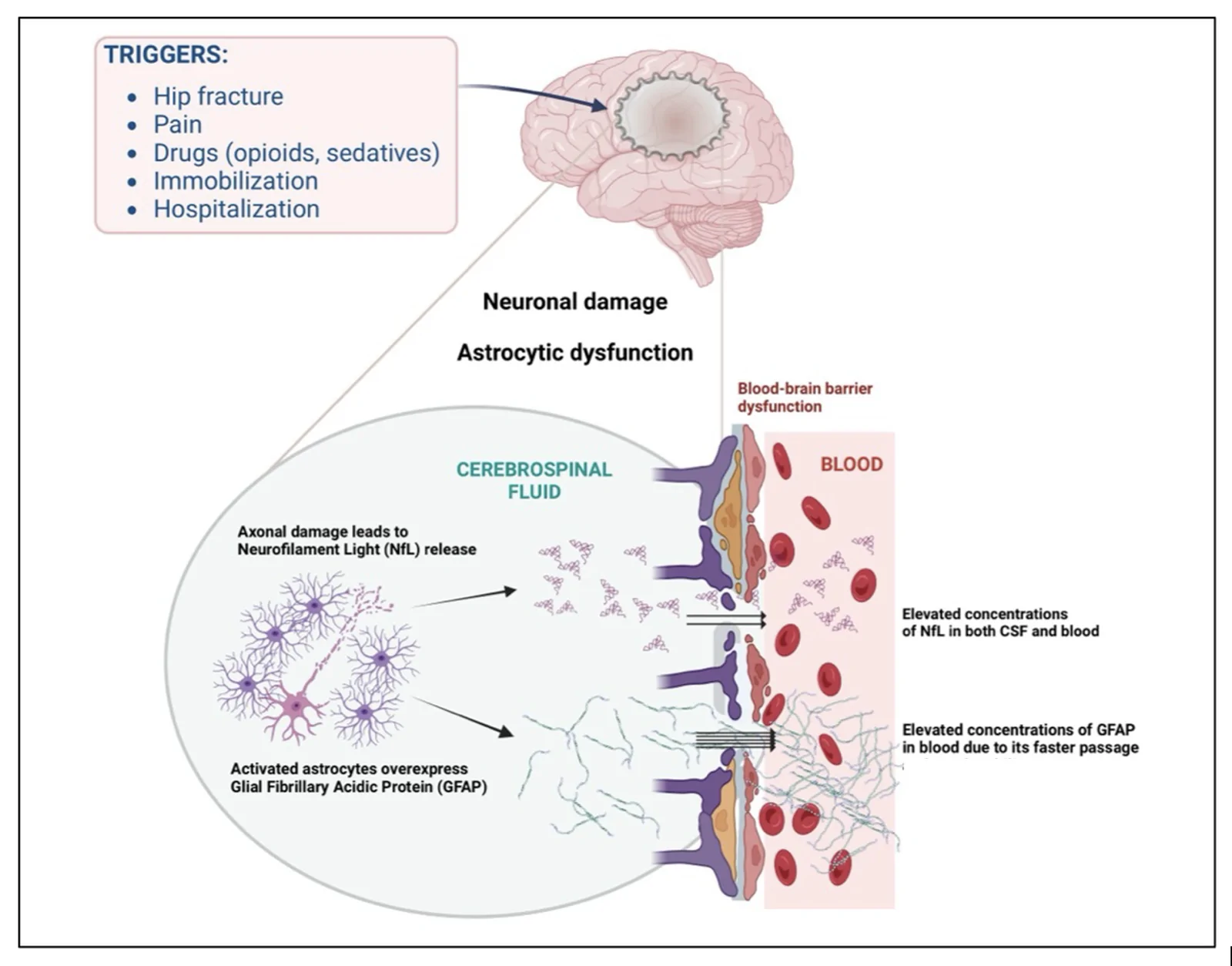
**Hypothetical pathophysiological mechanisms involving NfL and GFAP in POD occurrence.**Figure created with BioRender (https://www.biorender.com). Abbreviation: CSF = cerebrospinal fluid.

## DISCUSSION

This study shows that higher preoperative blood concentrations of NfL and GFAP are associated with increased odds of POD among older patients with HF, independent of age, sex, dementia, frailty, and preoperative IL-6 concentration.

Preoperative blood concentrations of NfL and GFAP were significantly higher in patients with HF who developed POD than in those who did not, and the results were confirmed after stratifying the population according to POD and dementia status. As for the intraoperative CSF, NfL, but not GFAP, concentrations were significantly higher in POD patients, although a significant correlation was found between NfL and GFAP concentrations in the CSF of the overall population.

In recent years, two systematic reviews of delirium biomarkers have been conducted, yielding inconclusive results [11, 12]. Among the potential BBMs proposed in the most recent study, neuroinflammatory mediators seemed to be the most consistent biomarkers, but the authors observed that these mediators are often elevated in older patients with sepsis, trauma, or surgery, and are not specific for delirium [11]. Consistent with this observation, IL-6 concentration was included as a potential confounder of the relationship in our study, but it was not found to be independently associated with POD. Accounting for IL-6 may be crucial to determining whether the association between neurodegeneration and POD is mediated by systemic inflammation-related pathways [37–39].

Our findings on NfL and GFAP contribute to the growing body of evidence supporting blood NfL as a valuable biomarker of neuroaxonal damage in delirium research while also providing new insights into the ongoing debate regarding GFAP as a potential BBM.

Currently, NfL has emerged as a sensitive marker for neuroaxonal damage shortly after brain injury, with higher concentrations in the blood or CSF of patients with delirium compared to those without it across medical wards [23, 24], Intensive Care Units [21, 40], and elective surgical contexts [13, 15, 22, 41–43]. Conversely, a recent nested case-control study involving 35 matched pairs of patients undergoing elective knee or hip replacement surgery did not find significant differences in blood NfL concentrations between the POD and Non-POD groups [44]. However, their findings may not accurately reflect the real-world population due to selective inclusion criteria and the categorization of subsyndromal delirium as full-syndromal delirium. Only one study investigated NfL concentrations in both blood and CSF in the older HF population, reporting significantly elevated serum levels —but not CSF levels— in patients with delirium [14]. Incomplete CSF sampling, heterogeneity in dementia status, and the inclusion of subsyndromal delirium and preoperative delirium cases, may have contributed to these discrepancies.

Similarly, increased GFAP expression is a hallmark of response to brain injury, related to astrocytic dysfunction, namely reactive astrogliosis [18, 20], but the relationship between GFAP and delirium is even more controversial than that between GFAP and NfL. According to previous studies, an elevated blood GFAP level was associated with POD in a population undergoing major cardiac and gastrointestinal surgery [22], but not in patients undergoing major elective non-intracranial non-cardiac surgery [43, 45], nor in a heterogeneous case-mix of delirious patients [23]. In the retrospective study by Piel et al. [23], delirium was identified exclusively through discharge diagnosis codes, without systematic in-hospital assessment, and dementia status among delirious patients was not evaluated. Leung et al. [43] retrospectively recruited patients who were mainly cognitively unimpaired, limiting generalizability to high-risk populations. Lastly, Ballweg et al. [45] analyzed data from two cohort studies excluding patients with diagnosed dementia, and the delirium group was relatively young (mean age=69 years), thereby limiting comparability with typical high-risk populations. Therefore, differences in study design and patient populations, along with the lack of adjustment for systemic inflammatory confounders [39, 46], are likely key factors contributing to the variability observed in previous findings. Moreover, reliance on single-fluid biomarker analyses and variability in analytical methods, such as differences in assay sensitivity and sample processing protocols, may contribute to the inconsistencies observed across studies. Notably, no study to date has specifically investigated the relationship between GFAP and POD in orthogeriatric settings, despite the particularly high risk of delirium in older adults undergoing urgent HF surgery.

The significant correlation between blood and CSF concentrations for NfL and GFAP supports the biological plausibility of our findings, which is consistent with the literature [14, 44]. This suggests that peripheral blood concentrations could serve as reliable surrogates for brain pathology, offering a non-invasive and cost-effective approach for large-scale clinical assessments compared to direct CSF sampling. Nevertheless, the lack of a significant correlation between GFAP blood and CSF in the POD group deserves commentary, which also applies to the absence of significant differences in GFAP CSF between POD and Non-POD groups. A potential explanation should consider the different clearance mechanisms of these markers in biological fluids. Previous studies demonstrated that blood GFAP concentration increases rapidly after orthopaedic trauma or traumatic brain injury and remains elevated for several days [47], and delirium is associated with astrocyte activation and increased blood-brain barrier (BBB) permeability [48], both of which could facilitate GFAP release in the blood [49]. In contrast, NfL turnover times in the blood and CSF are similar [50].

As for the pathophysiological mechanisms of early elevation of NfL and GFAP levels in older patients with POD, Figure 3 shows a hypothetical unifying pathway, integrating the existing literature on neuroaxonal injury and astrocytic activation in relation to acute neurological stress [51, 52]. Neuronal damage and reactive astrogliosis may be caused by several triggers in older HF patients (i.e., hip fracture with acute inflammation, pain, drugs, immobilization, and hospitalization), which results in neuroaxonal destruction and the release of cytoskeletal proteins into the CSF and bloodstream. Endothelial damage, frequently observed during episodes of delirium, compromises the integrity of the BBB, leading to increased permeability and facilitating substance transport. This hypothesis is consistent with the current understanding of delirium pathophysiology, which likely involves a combination of acute inflammation, BBB disruption, and neurodegeneration [53, 54]. The concurrent elevation of NfL and GFAP levels in patients who subsequently develop POD supports a multifaceted pathophysiological process involving both neuronal injury and glial activation [53]. This interplay may reflect a broader neuroinflammatory response that contributes to delirium onset. Preoperative elevation of NfL and GFAP levels could serve as useful BBMs for identifying patients at risk of developing postoperative delirium. However, further research is needed to clarify the underlying pathophysiological mechanisms and determine whether targeted interventions could modulate these BBMs and reduce the incidence of delirium.

Our findings also suggest that the risk of POD increases with elevated blood NfL and GFAP concentrations, regardless of dementia status. Individuals with both dementia and POD exhibited higher concentrations of these biomarkers than those without dementia or POD. This points to shared mechanisms between dementia and delirium, contributing to increased susceptibility to POD in patients with dementia. As both NfL and GFAP have been consistently associated with dementia development [55], it is plausible that the pathophysiological processes connecting them to delirium may contribute to a higher risk of long-term cognitive impairment following a delirium episode [56]. The differences observed in the BBMs concentrations across subgroups stratified by the presence or absence of POD and dementia could also be explained by this tripartite association.

Given the notable unmet diagnostic need for delirium, mirrored by a significant therapeutic gap in the field, BBMs such as NfL and GFAP could be crucial for the early recognition of at-risk patients or in cases of ambiguous diagnoses, together with potentially elucidating the underlying pathophysiological mechanisms of delirium. Multimodal prevention and therapeutic strategies may stem from BBMs identification to enhance patient outcomes.

The present study has several strengths. This is one of the few studies designed to investigate potential BBMs of POD in a large sample of older adults with HF, providing both blood and CSF data. From a methodological point of view, all biological samples were analysed simultaneously in the same laboratory by technicians blinded to the clinical data, ensuring consistency and reducing bias. Delirium assessment was rigorously performed using a well-known and validated tool. To address the impact of neuroinflammation and neurodegeneration, the presence of dementia and baseline inflammatory levels were treated as confounding factors.

Regarding limitations, the potential dementia detection bias should be considered, as a conclusive diagnosis of dementia was not always achievable. However, extensive chart reviews and structured questionnaires likely minimized the risk of false negatives. Reaching a consensus based on all available clinical information remains the most practical approach for this patient population, although early-stage pathological changes may have been overlooked in some older adults. Moreover, other potential biomarkers of neurodegeneration, such as amyloid beta and tau proteins or kynurenic acid, were not investigated because of the limited amount of biological material that could be collected, especially CSF, for both technical and ethical reasons. Lastly, the dichotomization of BBM values in the complete multivariable analysis may have led to a loss of data granularity, while the limited number of available CSFs may have affected the statistical power, particularly for GFAP. Future studies should validate these results using external datasets and possibly include larger CSF cohorts to further expand upon these findings.

### Conclusions

In summary, we found that higher preoperative blood concentrations of NfL and GFAP were significantly associated with POD occurrence in patients with HF, suggesting a link between blood-based detection of brain injury-related structural proteins and POD. While further research is needed to validate these findings, NfL and GFAP seem to offer insights into the biological underpinnings of delirium and might serve as minimally invasive BBMs for assessing POD risk in older patients undergoing HF surgery.

## Supplementary Materials

The Supplementary data can be found online at: www.aginganddisease.org/EN/10.14336/AD.2024.1676.

## Data Availability

Individual participant data cannot be made publicly available due to the sensitive nature of the personal health data collected and privacy and confidentiality reasons. However, under certain conditions, these data could be made accessible for statistical and scientific research. For further information, please contact the corresponding author.
