# Supplementary material for: Neurofilament-Light Chain and Glial Fibrillary Acidic Protein as Blood-Based Delirium Risk Markers: A Multicohort Study
Source: Aging Dis. 2025 Apr 18;17(3):1556–68. doi: 10.14336/AD.2025.0107 (PMC13061541; doi:10.14336/AD.2025.0107)
Supplement: Supplementary file 1 — The Supplementary data can be found online at: www.aginganddisease.org/EN/10.14336/AD.2024.1676. [file AD-17-3-1556-s.pdf]

SUPPLEMENTARY DATA

Neurofilament-Light Chain and Glial Fibrillary Acidic Protein as Blood-Based Delirium Risk Markers: A Multicohort Study

Maria Cristina Ferrara, Lucía Lozano-Vicario, Beatrice Arosio, Cristina D'Orlando, Lara De Luca, Alice Margherita Ornago, Elena Pinardi, Paolo Mazzola, Chukwuma Okoye, Riccardo Gamberale, Francesca Remelli, Massimiliano Castellazzi, Giovanni Zatti, Giuseppe Foti, Ángel Javier Muñoz-Vázquez, Nicolás Martínez-Velilla, Stefano Volpato, Giuseppe Bellelli on behalf of the ORTODEL group (Appendix 1)

© 2025. Ferrara MC. et al. Published online at http://www.aginganddisease.org/EN/10.14336/AD.2025.0107

SUPPLEMENTARY DATA

Supplementary Table 1. Key demographic and clinical characteristics of the two cohorts (ORTODEL and BIODEL).

Variables[a]	ORTODEL (*n*=83)	BIODEL (*n*=60)	p-value
Age	84 (76-88)	86 (81-91)	0.012
Sex (female)	60 (72.3)	50 (83.3)	0.122
Dementia	14 (16.9)	10 (16.7)	0.975
Clinical Frailty Scale	4 (3-6)	4 (3-5)	0.064
Charlson Comorbidity Index score	5 (4-6)	6 (4-7)	0.038
Time to surgery (days)	2 (1-2)	2 (1-2)	0.499
General anaesthesia	11 (13.3)	0 (0)	0.003
Preoperative blood IL-6[b] (pg/mL)	38.7 (22.1-60)	34.45 (23.65-48.95)	0.605
Preoperative blood NfL (pg/mL)	48.7 (28.9-78.5)	49.75 (38.6-79.2)	0.385
Preoperative blood GFAP (pg/mL) *(n=135)*	15.5 (10.4-22.2)	19.2 (11.2-24.1)	0.148
CSF[c] NfL[d] (pg/mL) *(n=106)*	1697.5 (1287.2-2188)	1734 (1173-2199)	0.944
CSF[c] GFAP[e] (pg/mL) *(n=104)*	546 (390-880)	602 (411-768)	0.969

[a] Nominal variables are expressed as n (%), while continuous variables are expressed as median (interquartile range); [b] IL-6 = Interleukin-6; [c] CSF = Cerebrospinal fluid; [d] NfL = Neurofilament-Light chain; [e] GFAP = Glial Fibrillary Acidic Protein

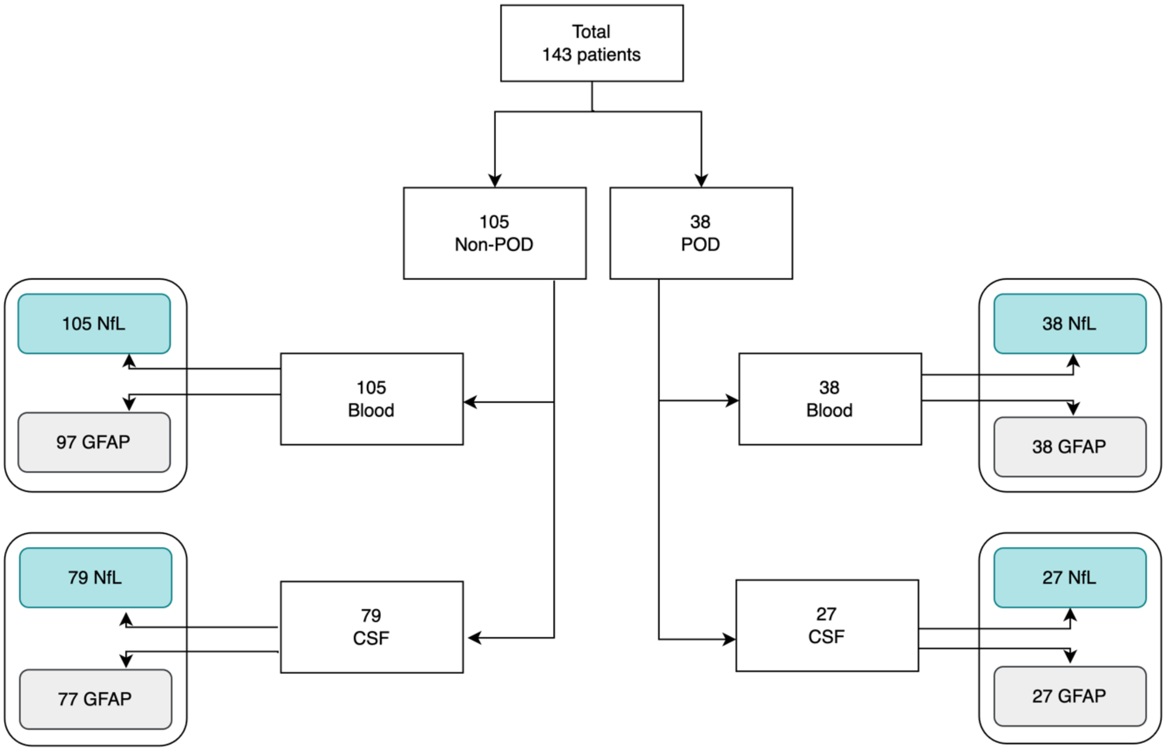

Supplementary Figure 1. Flowchart reporting the available samples of blood and CSF for NfL and GFAP analysis. POD = Postoperative delirium; NfL = Neurofilament-Light chain; GFAP = Glial Fibrillary Acidic Protein; CSF = Cerebrospinal fluid.

© 2025. Ferrara MC. et al. Published online at http://www.aginganddisease.org/EN/10.14336/AD.2025.0107

SUPPLEMENTARY DATA

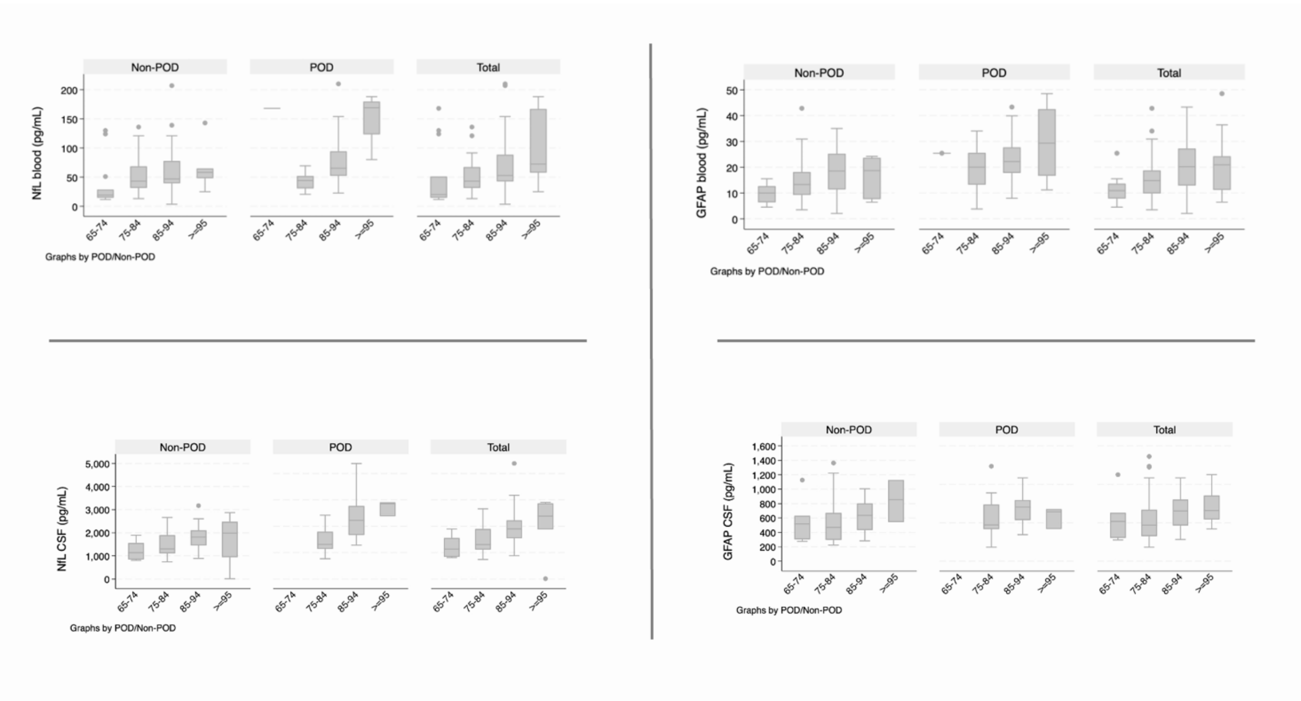

Supplementary Fig. 2. NfL and GFAP concentrations in the blood and CSF according to age group and within Non-POD and POD groups. POD = Postoperative delirium; NfL = Neurofilament-Light chain; GFAP = Glial Fibrillary Acidic Protein; CSF = Cerebrospinal fluid.

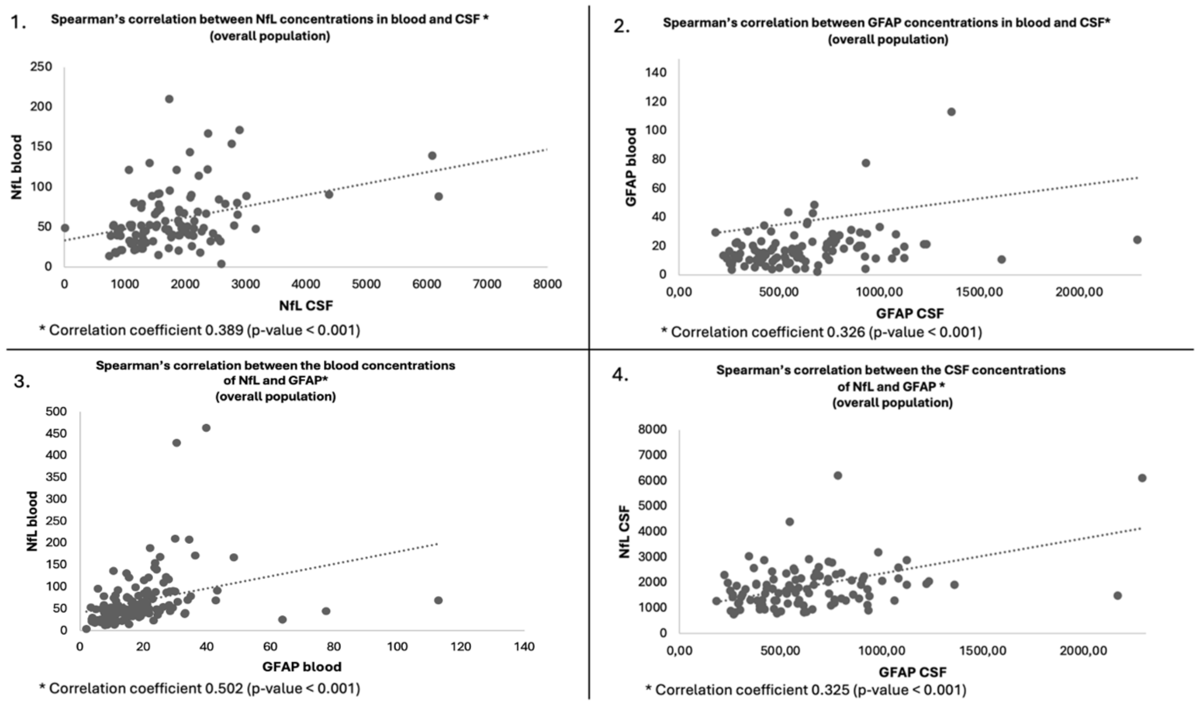

Supplementary Fig. 3. Intra-blood, intra-CSF and blood-CSF correlations in the overall population. NfL = Neurofilament-Light chain; GFAP = Glial Fibrillary Acidic Protein; CSF = Cerebrospinal fluid.

© 2025. Ferrara MC. et al. Published online at http://www.aginganddisease.org/EN/10.14336/AD.2025.0107

SUPPLEMENTARY DATA

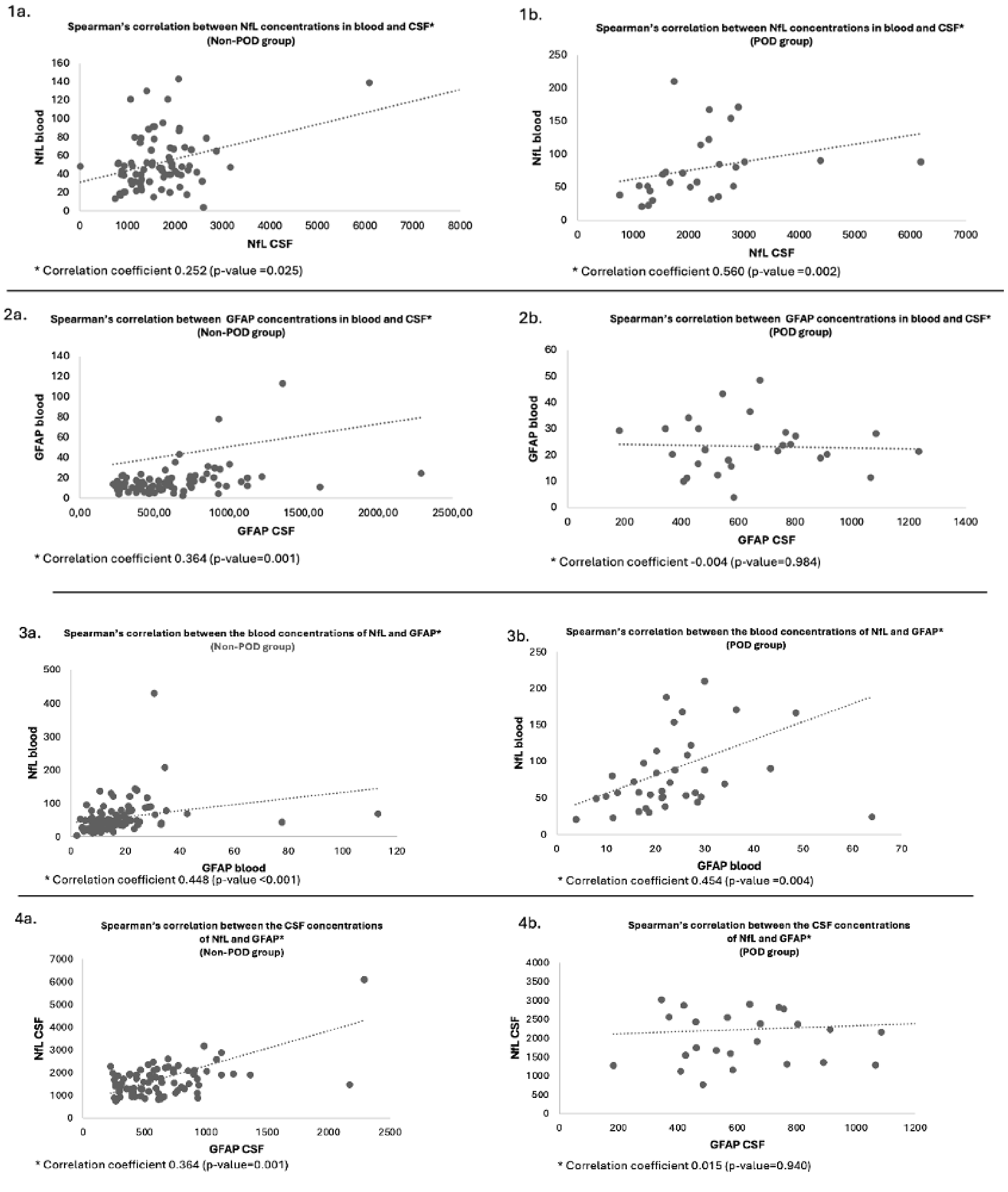

Supplementary Fig. 4. Intra-blood, intra-CSF and blood-CSF correlations in the overall population and within Non-POD and POD groups. POD = Postoperative delirium; NfL = Neurofilament-Light chain; GFAP = Glial Fibrillary Acidic Protein; CSF = Cerebrospinal fluid.

© 2025. Ferrara MC. et al. Published online at http://www.aginganddisease.org/EN/10.14336/AD.2025.0107

SUPPLEMENTARY DATA

Appendix 1. Collaborators of the ORTODEL Working Group.

Name	Location	Role	Contribution
Luca Tinelli	School of Medicine and Surgery University of Milano-Bicocca, Italy;	Site investigator	Collection of data
Alessio Greco	ASST Lecco, Italy	Site investigator	Collection of data
Alberto Finazzi	School of Medicine and Surgery University of Milano-Bicocca, Italy	Site investigator	Collection of data
Daniele Munegato	IRCCS Foundation San Gerardo dei Tintori, Italy	Site investigator	Collection of data
Gaetano Caruso	Department of Neurosciences and Rehabilitation, University of Ferrara, Italy;	Site investigator	Collection of data
Marco Sapia	Department of Medical Sciences, University of Ferrara, Italy;	Site investigator	Collection of data
Pierfederico Scaroni	Department of Medical Sciences University of Ferrara, Italy.	Site investigator	Collection of data

© 2025. Ferrara MC. et al. Published online at http://www.aginganddisease.org/EN/10.14336/AD.2025.0107
